# Benefits of Living Over Deceased Donor Kidney Transplantation in Elderly Recipients. A Propensity Score Matched Analysis of a Large European Registry Cohort

**DOI:** 10.3389/ti.2024.13452

**Published:** 2024-08-23

**Authors:** Néstor Toapanta, Jordi Comas, Ignacio Revuelta, Anna Manonelles, Carme Facundo, María José Pérez-Saez, Anna Vila, Emma Arcos, Jaume Tort, Magali Giral, Maarten Naesens, Dirk Kuypers, Anders Asberg, Francesc Moreso, Oriol Bestard, the EKITE consortium

**Affiliations:** ^1^ Kidney Transplant Unit, Nephrology Department, Vall d’Hebron University Hospital, Vall d’Hebron Research Institute (VHIR), Vall d’Hebron Barcelona Hospital Campus, Autonomous University of Barcelona, Barcelona, Spain; ^2^ Catalan Transplantation Organization, Barcelona, Spain; ^3^ Kidney Transplant Unit, Nephrology Department, Hospital Clinic, Barcelona, Spain; ^4^ Kidney Transplant Unit, Nephrology Department, Bellvitge University Hospital, Bellvitge Biomedical Research Institute (IDIBELL), Barcelona University (UB), Barcelona, Spain; ^5^ Kidney Transplant Unit, Nephrology Department, Fundació Puigvert, Barcelona, Spain; ^6^ Kidney Transplant Unit, Nephrology Department, Hospital del Mar, Barcelona, Spain; ^7^ Kidney Transplant Unit, Nephrology Department, Hospital Universitari Germans Trias i Pujol, Badalona, Spain; ^8^ CRTI UMR 1064, Inserm, Université de Nantes, ITUN, CHU Nantes, RTRS Centaure, Nantes, France; ^9^ Department of Microbiology, Immunology, and Transplantation, KU Leuven, Leuven, Belgium; ^10^ Department of Transplantation Medicine, Oslo University Hospital, Oslo, Norway; ^11^ Department of Pharmacy, University of Oslo, Oslo, Norway

**Keywords:** living donor, deceased donor, survival, elderly renal transplant, propensity score analysis

## Abstract

Although kidney transplantation from living donors (LD) offers better long-term results than from deceased donors (DD), elderly recipients are less likely to receive LD transplants than younger ones. We analyzed renal transplant outcomes from LD versus DD in elderly recipients with a propensity-matched score. This retrospective, observational study included the first single kidney transplants in recipients aged ≥65 years from two European registry cohorts (2013–2020, n = 4,257). Recipients of LD (n = 408), brain death donors (BDD, n = 3,072), and controlled cardiocirculatory death donors (cDCD, n = 777) were matched for donor and recipient age, sex, dialysis time and recipient diabetes. Major graft and patient outcomes were investigated. Unmatched analyses showed that LD recipients were more likely to be transplanted preemptively and had shorter dialysis times than any DD type. The propensity score matched Cox’s regression analysis between LD and BDD (387-pairs) and LD and cDCD (259-pairs) revealing a higher hazard ratio for graft failure with BDD (2.19 [95% CI: 1.16–4.15], *p* = 0.016) and cDCD (3.38 [95% CI: 1.79–6.39], *p* < 0.001). One-year eGFR was higher in LD transplants than in BDD and cDCD recipients. In elderly recipients, LD transplantation offers superior graft survival and renal function compared to BDD or cDCD. This strategy should be further promoted to improve transplant outcomes.

## Graphical Abstract

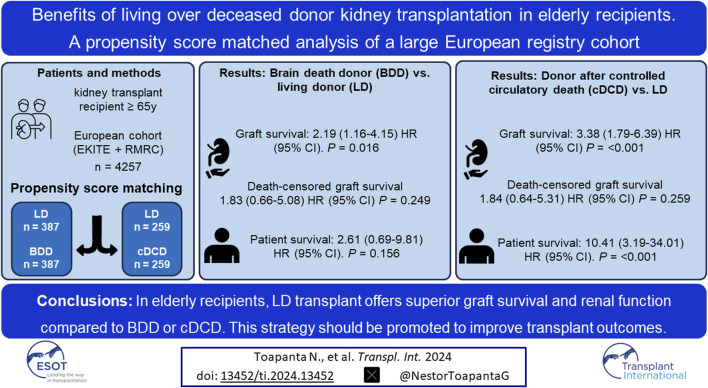

## Introduction

In recent decades, a growing number of elderly patients with end-stage kidney disease (ESKD) have needed to start renal replacement therapy [1–3]. Although kidney transplantation (KT) has been shown to offer better survival and quality of life than dialysis in elderly patients [4–8], some studies have questioned these benefits, especially for those receiving extended criteria donor grafts after circulatory death (DCD). In this sense, using data from the Dutch Organ Transplantation Registry, Peters-Sengers et al. reported that only 40% of elderly (≥65 years) recipients of elderly DCD transplants were alive with a functioning graft at 5 years compared with 53% of elderly recipients of elderly brain death donors (BDD) and 61% of elderly recipients from young donors. Notably, the authors also showed that this group of elderly recipients of elderly kidneys obtained from DCD had a 5-year mortality rate comparable to that of waitlisted elderly patients who remained on dialysis [9]. Similarly, our group recently described in a large European multicenter cohort, a significantly higher rate of graft loss among recipients of extended criteria controlled DCD (cDCD) (9.5 per 1,000 recipient-month [95% CI 6.8–12.7]) compared with recipients of extended criteria BDD (5.2 per 1,000 recipient-month [95% CI 4.2–6.3] or recipients of standard criteria donors (1.8 for standard BDD and 2.8 per 1,000 recipient-month for standard cDCD) [10]. Taken together, these results raise the question of whether highly extended kidneys should be assigned to similarly extended recipients, particularly if a DCD kidney transplant is employed.

Living donor (LD) kidney transplantation has been widely associated with superior graft and patient survival compared with deceased donor (DD) kidney transplantation in patients with ESKD [11]. However, information is scarce about the results of LD kidney transplantation in the elderly population. Along these lines, Berger et al. carried out a study of 219 LD kidney transplant recipients aged ≥70 and observed a greater graft loss as compared with LD aged 50–59 years (subhazard ratio 1.62), but not different from matched 50-to 59-year-old DD allografts without extended criteria. Importantly, mortality in LD aged ≥70 years was not higher than in matched healthy controls included in the NHANES III study [12]. Recently, Tegzess et al. conducted a retrospective single-center study of 348 elderly kidney transplants (median age 68 years [66–70]) performed between 2005 and 2017 and showed that recipients from an LD displayed a higher 5-year death-censored graft survival than recipients from the regular allocation (ETKAS) and the Euro-transplant Senior Program (ESP) (97.7% vs. 88.1% vs. 85.6; *p* < 0.001). Importantly, although the proportion of patients who received a preemptive kidney transplant was much higher in the LD cohort (60%) than in the other groups (11% and 13%), the authors did not observe any significant benefit in 5-year patient survival (71.7% vs. 67.4% vs. 61.9%, *p* = 0.480) [13].

To further characterize the benefits of LD compared with DD in the current era, we conducted a retrospective study in a large European cohort comprising 4,257 consecutive renal transplant patients to analyze graft outcomes in elderly transplant recipients (≥65 years) who received a kidney organ from LD, BDD or cDCD between 2013 and 2021. Importantly, to overcome the unbalanced nature of the different groups for some relevant variables (preemptive transplants, time on dialysis and recipient comorbidities), we performed a propensity score analysis to accurately match the different study populations. To increase the statistical power of our analysis we analyzed data from two well-characterized European renal transplant Registries.

## Patients and Methods

### Patients

For the present study we combined data on patients from two European transplant registries: 1) The Catalan Registry of Renal Patients (RMRC; approved by the Catalan Government; DOGC 402, 27 January 1984) which is a mandatory population-based registry of renal patients covering 7.5 million inhabitants that collects information from all patients with End Stage Renal Disease requiring Renal Replacement Therapy (www.trasplantaments.gencat.cat). This registry includes clinical data from all adult kidney transplant units in Catalonia: Hospital Universitari Vall d’Hebron, Hospital Clinic, Hospital Universitari Bellvitge, Fundació Puigvert, Hospital del Mar and Hospital Universitari Germans Trias i Pujol). 2) the EKITE cohort (approved by the CNIL, n°917155) [14] including data from seven European transplant centers from France (Nantes, Nancy, Lyon, Montpellier, Nice), Norway (Oslo) and Belgium (Leuven) since 2013 and merged into a single European cohort updated annually. All first kidney transplants from LD or DD, either BDD or cDCD aged 65 years or older, from January 2013 to December 2021, were considered for the present study. Recipients from uncontrolled donors after circulatory death were excluded. Patients were followed up until 31 December 2021. Baseline donor (age and type) and recipient variables (age, sex, time on dialysis, diabetes, cardiovascular disease) were recorded. Outcomes focused on graft survival, death-censored graft survival, patient survival and renal function.

Additionally, through the RMRC we gathered information on 95.4% (155/159) kidney donors from recipients over 65 years of age from 2013 to 2021, with follow-up until 31 December 2021.

The reported clinical and research activities adhere to the Declaration of Helsinki and are consistent with the Principles of the Declaration of Istanbul as outlined in the Declaration of Istanbul on Organ Trafficking and Transplant Tourism.

### Statistical Analysis

Variables were described as mean ± standard deviation, median and interquartile range, or frequencies according to their distribution. Qualitative variables were compared by the Chi-squared test, non-normally distributed quantitative variables by the Kruskal-Wallis test and normally distributed quantitative variables by the analysis of variance (ANOVA). Kaplan-Meier analysis was employed to calculate survival curves and the log-rank test was used for comparisons. Univariate and multivariable Cox’s regression analysis was employed after verifying its proportionality to estimate risks.

Propensity score matching without replacement was employed to define a cohort of paired cases (recipients of LD vs. BDD and recipients of LD vs. cDCD) by age (donor and recipient), sex, time on renal replacement therapy before transplantation and diabetes mellitus. Cardiovascular disease was excluded from matching due to the presence of missing data (n = 72).

A two-tailed *p*-value <0.05 was considered significant and STATA17.0 was employed for statistical analysis.

## Results

### Donor and Recipient Characteristics

**FIGURE 1:**
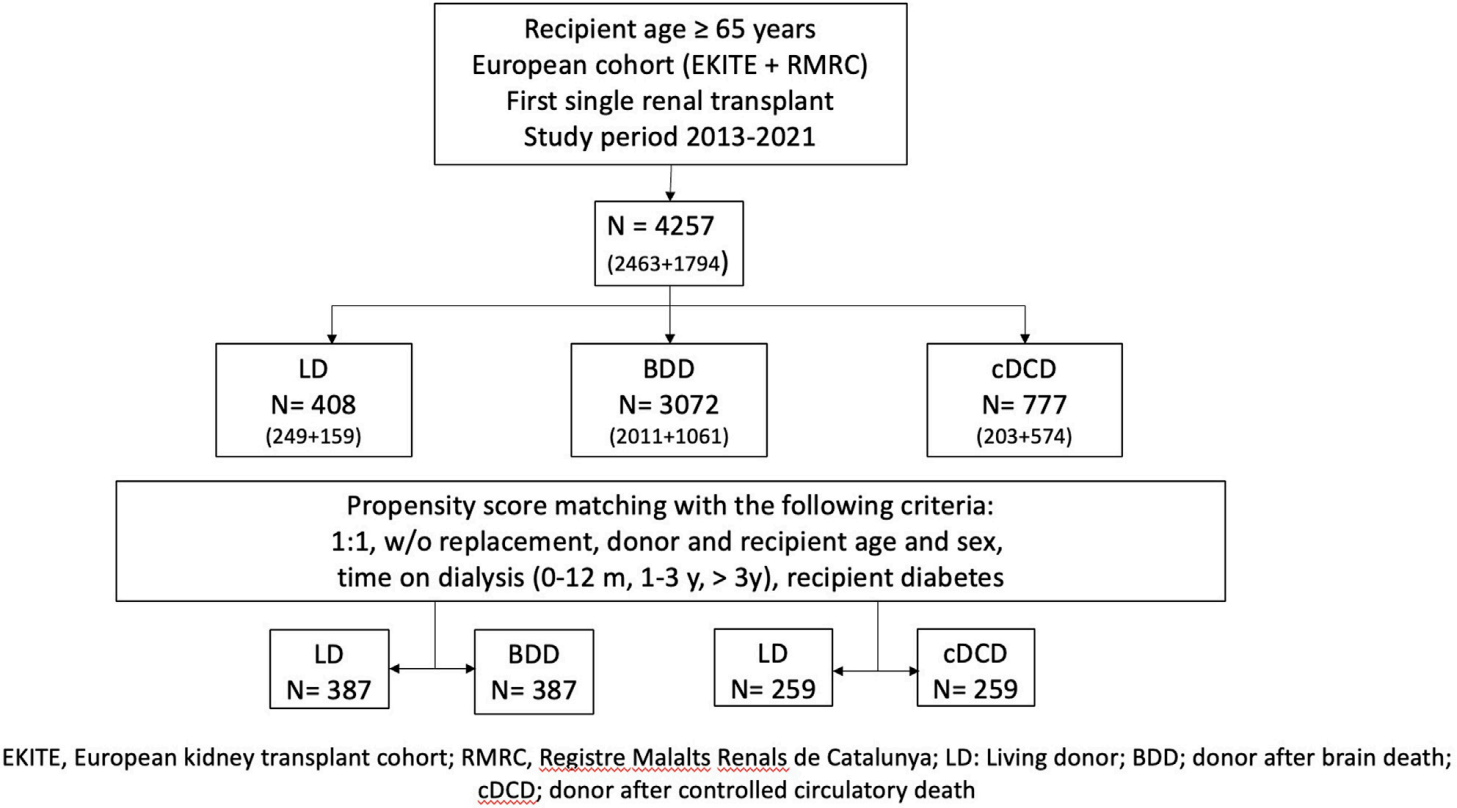
Flow-chart of the included population.

**FIGURE 2:**
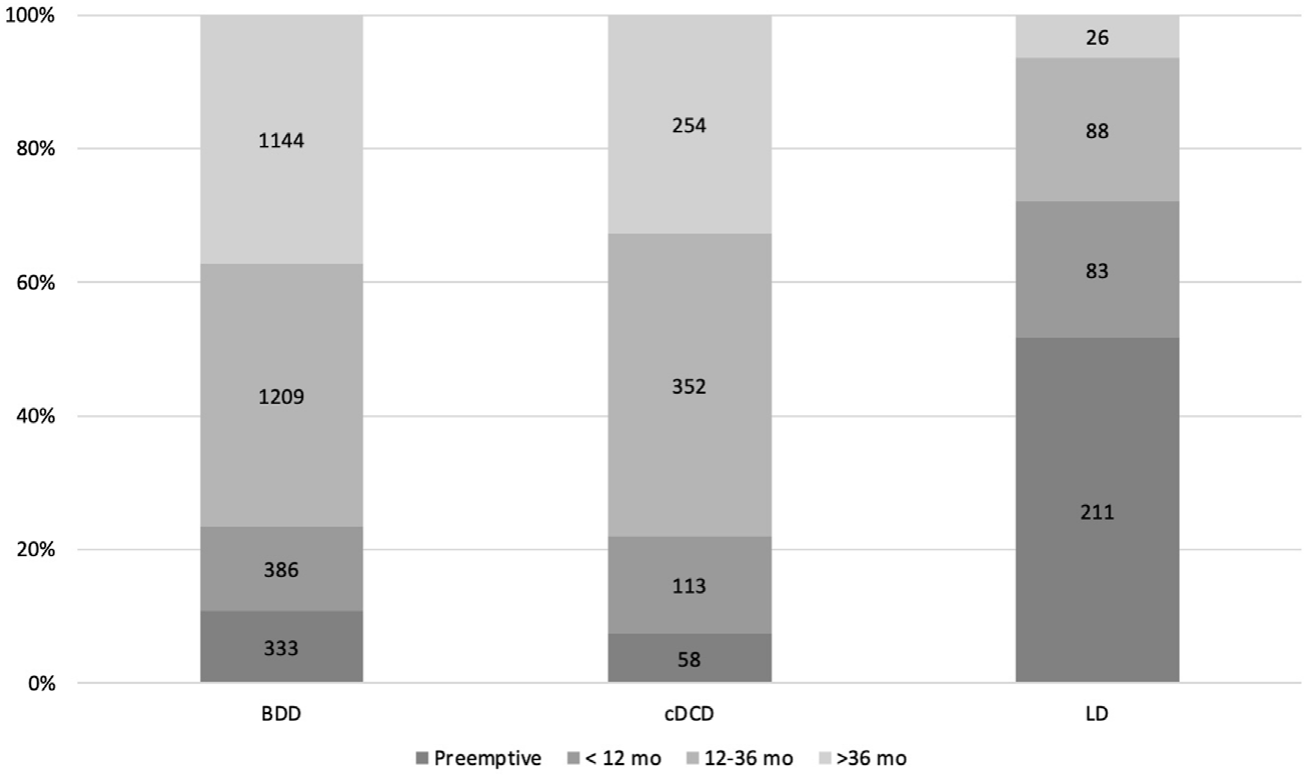
Distribution of time on dialysis across the different donor sources. LD, living donor; DBD, donors after brain death; cDCD, donor after controlled circulatory death.

This European study cohort included 4,257 consecutive, adult, single KT from LD (n = 408), BDD (n = 3,072) and cDCD (n = 777) (Figure 1). Baseline donor and recipient characteristics are displayed in Table 1. The mean donor and recipient age and dialysis vintage were lower in LD than in BDD and cDCD. Male recipients were more frequent in LD, while there were fewer LD patients with diabetes and cardiovascular disease. Time on dialysis was shorter in the case of LD and a higher percentage were transplanted pre-emptively (51.7%) as compared to BDD (10.8%) and cDCD (7.4%) (Figure 2). Regarding blood groups, A and O were the most common among the three groups. The time on dialysis was particularly long for patients with blood group O, while approximately 50% of DD transplants were on dialysis for more than 3 years before receiving a kidney transplant, only 20% of blood group A patients were on dialysis for more than 3 years before receiving a DD organ. Conversely, LD kidney transplants were much less likely to spend more than 3 years on dialysis across all blood groups (4.4% and 9.2% for blood groups A and O, respectively).

**TABLE 1:** Donor and recipient characteristics of renal transplants from the BDD, cDCD, and LD cohorts.

Variables	BDD (n = 3,072)	cDCD (n = 777)	LD (n = 408)	P
Age of donors, years	71.5 ± 9.8	67.2 ± 11.1	59.2 ± 11.2	<0.001
Age of recipient, years	71.4 ± 4.4	70.6 ± 4.4	69.4 ± 3.3	<0.001
Male sex, %	66.2	67.4	77.9	<0.001
Time on dialysis, Pre-emptive/0–12 mo./1–3 y/>3 y, %	10.8/12.5/39.3/37.2	7.4/14.5/45.3/32.6	51.7/20.3/21.5/6.3	<0.001
Blood group A/B/AB/0, %	45.2/10.3/4.7/39.7	44.7/8.1/2.9/44.1	50.1/9.6/2.7/37.4	0.016
Blood group A and time of dialysis 0–12 mo./1–3 y/>3y	32.8/45.2/21.9	31.7/49.4/18.8	78.2/17.3/4.4	<0.001
Blood group B and time of dialysis 0–12 mo./1–3 y/>3y	26.0/33.3/40.6	24.5/44.2/31.1	69.2/25.6/5.1	<0.001
Blood group AB and time of dialysis 0–12 mo./1–3 y/>3y	42.1/39.8/18.0	45.4/31.8/22.7	81.8/9.09/9.09	0.144
Blood group 0 and time of dialysis 0–12 mo./1–3 y/>3y	13.2/37.6/49.1	11.5/42.4/46.0	62.9/27.8/9.2	<0.001
Diabetes, %	42.9	44.2	41.4	0.635
Cardiovascular disease, %	57.8	59.7	44.3	<0.001

LD, Living donors; DBD, donors after brain death; cDCD, donors after controlled circulatory death.

### Survival Analysis Without Propensity Score

**FIGURE 3:**
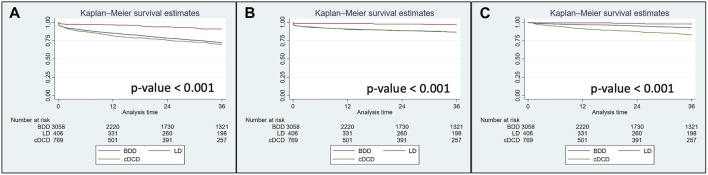
Graft survival including graft failure and patient death with functioning graft **(A)**, death-censored graft survival **(B)** and patient survival censoring after graft loss **(C)** in kidney transplants performed during 2013–2021 in the European cohort. Log-rank *p*-value for all comparisons is displayed. LD, living donors; BDD, donors after brain death; cDCD, donor after controlled circulatory death.

Univariate Kaplan-Meier analysis showed that 3-year graft survival including death with a functioning graft as well as both death-censored graft survival and patient survival were significantly higher in LD recipients than in BDD and cDCD recipients (Figures 3A–C). As shown in Tables 2, 3, multivariable Cox’s regression analyses adjusting for confounding variables such as donor and recipient age >70 years old, sex and relevant recipient comorbidities, confirmed these data for graft survival and death-censored graft survival. For patient survival censored after graft loss, univariate and multivariable analysis showed this similar trend (hazard ratios [95% confidence interval] of 3.03 [0.93–9.84], *p* = 0.066 and 11.34 [3.37–38.21], *p* < 0.001, for LD vs. BDD and LD vs. cDCD, respectively).

**TABLE 2:** Univariate and multivariate Cox’s regression analysis comparing outcomes in living donor (LD) and donor after brain death (DBD) kidney transplantation.

	Univariate Cox’s regression		Multivariate Cox’s regression	
DBD vs. LD	HR (95% CI)	*p*-value	HR (95% CI)	*p*-value
Graft survival	3.53 (2.43–5.11)	<0.001	2.64 (1.64–4.50)	<0.001
Death-censored graft survival	4.87 (2.60–9.13)	<0.001	2.59 (1.19–5.67)	0.017
Patient survival	3.33 (1.56–7.10)	0.002	3.03 (0.93–9.84)	0.066

Variables included in the multivariate analysis were donor age >70y, recipient age >70 y, recipient sex, recipient comorbidities (diabetes, cardiovascular disease) and time on dialysis.

**TABLE 3:** Univariate and multivariate Cox’s regression analysis comparing outcomes in living donor (LD) and donor after controlled circulatory death (cDCD) kidney transplantation.

	Univariate Cox’s regression		Multivariate Cox’s regression	
cDCD vs. LD	HR (95% CI)	*p*-value	HR (95% CI)	*p*-value
Graft survival	3.97 (2.69–5.67)	<0.001	3.90 (2.15–7.06)	<0.001
Death-censored graft survival	4.90 (2.54–9.44)	<0.001	3.06 (1.27–7.39)	0.013
Patient survival	8.16 (3.78–17.60)	<0.001	11.35 (3.37–38.21)	<0.001

Variables included in the multivariate analysis were donor age >70 y, recipient age >70 y, recipient sex, recipient comorbidities (diabetes, cardiovascular disease) and time on dialysis.

### Propensity Score Matching

After propensity score matching, we obtained 387 pairs of recipients from LD and BDD and 259 pairs of recipients from LD and cDCD. Baseline donor and recipient characteristics are displayed in Tables 4, 5, respectively. As shown, the proportion of preemptive transplantations and the time on dialysis were now well matched between pairs from both cohorts (Supplementary Figures S1A, B).

**TABLE 4:** Baseline donor and recipient characteristics with propensity score matching between LD and DBD.

Variables	BDD (n = 387)	LD (n = 387)	P
Age of donors, years	60.9 ± 13.6	60.3 ± 10.3	0.468
Age of recipients, years	69.6 ± 3.7	69.6 ± 3.3	0.740
Recipient sex (m/f), %	80.6/19.3	76.7/23.2	0.188
Time on dialysis, Pre-emptive/0–12 mo./1–3 y/>3 y, %	44.9/21.1/25.8/8.0	49.3/21.4/22.4/6.7	0.544
Diabetes, %	42.8	43.9	0.191
Cardiovascular disease, %	48.7	43.9	0.191

LD, Living donors; BDD, donors after brain death; DM, diabetes mellitus.

**TABLE 5:** Baseline donor and recipient characteristics, with propensity score matching between LD and cDCD.

Variables	cDCD (n = 259)	LD (n = 259)	P
Age of donors, years	60.5 ± 12.9	61.6 ± 10.6	0.284
Age of recipients, years	69.4 ± 3.8	69.9 ± 3.4	0.122
Recipient sex (m/f), %	77.6/22.3	72.2/27.8	0.156
Time on dialysis, Pre-emptive/0–12 mo./1–3 y/>3 y, %	21.6/31.2/37.1/10.0	25.8/30.5/33.5/10.0	0.694
Diabetes, %	36.6	43.2	0.127
Cardiovascular disease, %	52.2	47.5	0.300

LD, Living donors; cDCD, donors after controlled circulatory death; DM, diabetes mellitus.

**FIGURE 4:**
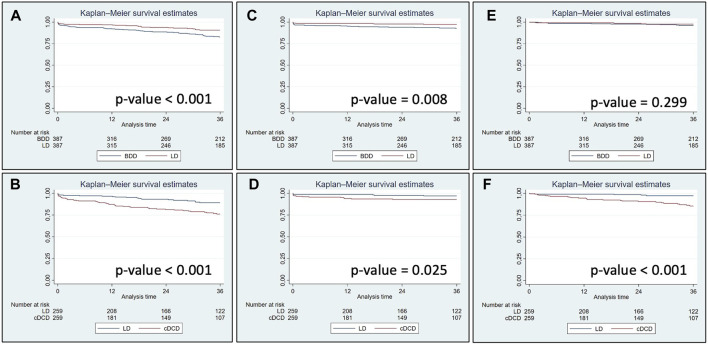
Graft survival including patient death **(A, B)**, death-censored graft survival **(C, D)** and patient survival **(E, F)** in kidney transplants performed during 2013–2021 in the European cohort matched by the propensity score. Log-rank *p*-value for all comparisons is displayed. LD, living donors; BDD, donors after brain death; cDCD, donors after controlled circulatory death.

Univariate Kaplan-Meier analysis showed that 3-year graft survival (including death with a functioning graft) and death-censored graft survival were significantly higher in LD recipients than in BDD and cDCD recipients (Figures 4A–D). However, patient survival censored for graft loss was not significantly different between LD and BDD recipients (Figure 4E) but was significantly lower in cDCD recipients than in LD recipients (Figure 4F). Adjusted multivariable Cox’s regression analysis showed that graft survival was higher in LD recipients in both paired cohorts, whereas death-censored graft survival was not significantly different between groups (Table 6). Moreover, patient survival in the matched populations when censored for graft loss displayed a very high risk for cDCD vs. LD (hazard ratio: 10.41 [3.19–34.01], *p*-value <0.001) while this risk did not reach statistical significance for BDD (hazard ratio: 2.61 [0.69–9.81], *p*-value = 0.156).

**TABLE 6:** Multivariable Cox’s regression analysis in patients evaluated by the propensity score matching.

	BDD (n = 387)	LD (n = 387)		cDCD (n = 259)	LD (n = 259)	
	HR (95% CI)		*p*-value	HR (95% CI)		*p*-value
Graft survival	2.19 (1.16–4.15)		0.016	3.38 (1.79–6.39)		<0.001
Death-censored graft survival	1.83 (0.66–5.08)		0.249	1.84 (0.64–5.31)		0.259
Patient survival	2.61 (0.69–9.81)		0.156	10.41 (3.19–34.01)		<0.001

Variables included in the multivariate analysis were donor age >70 y, recipient age >70 y, recipient sex, recipient comorbidities (diabetes, cardiovascular disease) and time on dialysis. BDD, brain death donors; LD, living donors; cDCD, donors after controlled circulatory death.

### Kidney Allograft Function

**FIGURE 5:**
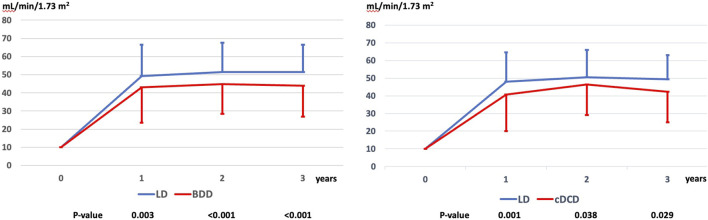
Evolution of renal function (eGFR according to the CKD-EPI formula) up to 3 years in the matched cohorts. LD, living donor; BDD, donors after brain death; cDCD, donor after controlled circulatory death; eGFR, estimated Glomerular Filtration Rate by the CKD-EPI formula.

The estimated glomerular filtration rate (eGFR) from 1 to 3 years of follow-up was significantly higher in LD as compared to BDD and cDCD and was already higher at 12 months after transplantation (Figure 5).

### Kidney Donor Evolution

**FIGURE 6:**
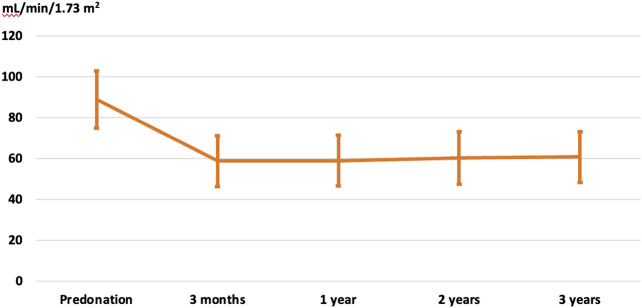
Evolution of renal function in kidney donors after nephrectomy (eGFR according to the CKD-EPI formula).

Data were available for 155 cases out of 159 living kidney donors employed to transplant elderly recipients from the RMRC. The mean age of the donors at the time of donation was 62.8 ± 8.9 years (range 36–78), female sex predominated (77.4%) and among the most relevant comorbidities were arterial hypertension (27.4%), dyslipidemia (30%), obesity (19.3%) and urolithiasis (3.8%). After nephrectomy, comorbidities remained stable (arterial hypertension in 15.8%, dyslipidemia in 29.1% and obesity in 7.9%) while a minority developed new-onset diabetes mellitus (1.3%). Notably, renal function remained stable after nephrectomy at 3 years (Figure 6).

## Discussion

We conducted a retrospective study of two large European cohorts of elderly renal transplant recipients to evaluate the benefits of receiving a graft from an LD versus a BDD or cDCD. Because these recipient populations were unbalanced for key clinical variables, we performed a propensity score analysis to match our populations. The results of our study confirm that LD offer advantages over DD (BDD or cDCD) in terms of graft survival including patient death and the need to return to dialysis. The propensity score analysis shows that the adjusted hazard ratio of graft failure in BDD recipients is more than twice that of LD, while it is more than three times that of cDCD. Because the rate of graft dysfunction after the first year is a low-frequency event in these matched cohorts, the adjusted model did not show significant differences in death-censored graft survival. Importantly, renal function was significantly higher in LD transplant recipients than in BDD or cDCD recipients, a key surrogate variable predicting long-term graft and patient outcomes [15]. More importantly, elderly LD transplant recipients are more likely to be transplanted preemptively and more quickly than both cDCD and DBD recipients.

The demographic profile of the ESKD population has changed over the last century, with older patients (≥65 years) representing the fastest-growing incident group starting maintenance dialysis therapy in developed countries [16, 17]. In parallel, elderly recipients have been progressively included in all kidney transplant programs in the United States and Europe [18]. In the present century, the number of elderly ESKD patients receiving a renal allograft has increased worldwide, changing in our geographical area from 12.3% of all renal transplants in 2000 to 38.2% in 2021 [19]. Therefore, there is an increasing interest in the outcome of transplantation in this cohort, as the proportion of older patients will gain significantly in terms of quality and quantity of life with successful kidney transplantation [5, 6, 20]. Although the outcomes of kidney transplantation from LD consistently exceed those from DD in terms of patient and graft survival [21], the opportunity for kidney transplantation from an LD is inconsistent across age categories. In the UK the likelihood of having an LD transplant rather than a DD transplant is almost 90% lower in those older than 65 years at the time of transplant, compared to young adults [22]. Similarly, in our country, the rate of LD kidney transplantation during the study period (2013–2021) was much lower in elderly recipients (8.8%) than in younger ones (24%).

In this study, one of the main differences between elderly KT receiving grafts from LD or DD is related to the time on dialysis. Importantly, more than 50% of LD received a pre-emptive KT while less than 10% of DD kidney transplants were performed before starting dialysis. The Descartes working group and the European Renal Best Practice (ERBP) Advisory Board recommend (grade 1D) that programs for pre-emptive kidney transplantation with LD kidneys should be encouraged [23]. However, they acknowledged a high risk of bias in their meta-analysis because patients selected for pre-emptive transplantation differed from those who were not. Patients receiving a pre-emptive transplant are more likely to receive a kidney from an LD and there were significant differences in comorbidities, socio-economic conditions, and education levels. A more recent meta-analysis including 76 studies comprising more than 120,000 patients confirmed the benefits of pre-emptive KT in terms of patient (adjusted HR: 0.78 [95% CI 0.66–0.92]) and death-censored graft survival (adjusted hazard ratio 0.81 [0.67–0.98]) [24]. However, as discussed well by the authors, the lead-time bias (e.g., the time difference in ESKD period in patients transplanted pre-emptively vs. those transplanted on dialysis) was not resolved by their meta-analysis. To overcome these limitations, we performed a propensity score matching to compare outcomes in kidney transplant recipients from LD donors vs. BDD or cDCD donors. The obtained cohorts (387 and 256 pairs, respectively) were well-matched for pre-emptive transplantation rates and dialysis duration, avoiding lead-time bias. Additionally, other key factors influencing patient and graft outcomes like donor and patient age, or patient comorbidities (diabetes) were also balanced in both cohorts. The propensity score-matched kidney transplant outcomes show that the adjusted hazard ratio for graft failure is more than twofold (hazard ratio 2.19 [95% CI 1.16–4.15]) for BDD recipients while it is more than threefold (hazard ratio 3.38 [95% CI 1.79–6.39]) for cDCD recipients. Notably, these differences were observed even though “very old” donors (>75 years) were not included in our propensity score analysis as this type of donor was much less represented in the LD cohort. In fact, the mean donor age in the matched cohorts was approximately 60 years, a figure very close to the mean donor age of deceased donors in our RMRC registry (58.6 years in 2021 and 60.8 years in 2020) [19]. Thus, our results confirm the benefit of LD kidney transplantation in the elderly population although we cannot estimate the potential benefit for elderly patients receiving highly extended DD kidneys. In this regard, data from the U.S. registry showed that recipients of older LD (≥65 years) have increased graft failure and long-term mortality compared to cases of younger LD; however, these recipients appear to do as well or better than recipients of standard or extended criteria deceased donors [25].

The number of KT with cDCD donors has exponentially increased in different countries in recent years, with a parallel increase in donor and recipient acceptance criteria. Although the outcomes of KT form cDCD have been reported to be comparable to those of BDD, studies in elderly recipients have yielded contradictory results [7, 9]. In the present study graft survival of kidney transplants from cDCD was lower than graft survival from BDD and patient death with a functioning graft is the major contributing factor to this finding (relative risk 10.6). Recently, data from the UK registry have shown that delayed graft function of more than 14 days in cDCD donors is associated with almost double the risk of patient death [26]. Although the presence of delayed graft function and its duration were not evaluated in our study, the high mortality risk in cDCD versus BDD recipients is consistent with a previous study conducted in patients from a large European patient cohort [10]. Management of cDCD donors for organ retrieval and organ preservation was also not recorded in our study. The benefits of normothermic regional perfusion over rapid recovery technique have been described in different studies [27–29] and the benefits of organ perfusion with different devices after retrieval over static cold storage have also been described, especially for kidney transplantation with long cold ischemia time [30].

An in-depth analysis of living donor outcomes is beyond the scope of the present study, but data from a subset of donors in this study confirm that renal function remains stable over the mid-term while major comorbidities (arterial hypertension, dyslipidemia, and obesity) are well controlled in this cohort of patients managed by transplant physicians.

Our study has some limitations because the data come from two large European transplant registries, and thus, detailed granularity on patient outcomes (e.g., cause of death) and graft outcomes (e.g., delayed graft function) was not available. However, the propensity score-matched analysis performed counterbalanced this constraint and allowed for accurate comparisons regarding the key hard outcomes investigated. Importantly, the mean donor age in the unmatched BDD and cDCD cohorts was close to 70 years, while after propensity score matching, the mean donor age dropped to 60 years, as “very old” donors were less frequently represented in the LD cohort. However, these donors are more easily found in this elderly patient population and are an optimal source for transplantation. Additionally, our findings are subject to residual confounding due to the lack of data on cardiovascular disease and other unmeasured factors such as social support and socioeconomic status. These factors, along with frailty, smoking, treatment adherence, and lifestyle, may influence graft and patient survival. Furthermore, we did not adjust or match for transplant variables such as HLA mismatch, which may differ between the LD and DD populations. Another limitation is that these results may not be generalizable to other organ allocation systems. In certain regions, kidneys from older and higher-risk donors are prioritized for elderly recipients, which could lead to a greater disparity between LD and DD compared to systems that do not impose such allocation restrictions.

In conclusion, our study strongly supports that LD transplantation offers significant advantages for elderly transplant recipients in terms of elective surgery, timely transplantation, graft survival and mid-term graft function. Thus, transplant teams should offer this treatment to elderly kidney transplant candidates to avoid the age-based inequity in access to transplantation [31].

## Data Availability

The raw data supporting the conclusions of this article will be made available by the authors, without undue reservation.

## Group Members of EKITE Consortium

**Oslo:** Karsten Midtvedt, Anna V Reisæter, Trond Jenssen, Kristian Heldal, AA. **Leuven:** Dirk Kuypers, MN, Borefore Jallah. **Lyon E. Hériot:** Lionel Badet, Maria Brunet, Fanny Buron, Rémi Cahen, Ricardo Codas, Sameh Daoud, Valérie Dubois, Coralie Fournie, François Gaillard, Arnaud Grégoire, Alice Koenig, Charlène Lévi, Emmanuel Morelon, Claire Pouteil-Noble, Maud Rabeyrin, Thomas Rimmelé, Olivier Thaunat. **Montpellier:** Nicolas Abdo, Sylvie Delmas, Moglie Le Quintrec, Vincent Pernin, Hélène Perrochia, Jean-Emmanuel Serre, Ilan Szwarc. **Nancy:** Alice Aarnink, Asma Alla, Pascal Eschwege, Luc Frimat, Sophie Girerd, Jacques Hubert, Raphaël Kormann, Marc Ladriere, François Lagrange, Emmanuelle Laurain, Pierre Lecoanet, Jean-Louis Lemelle; Anthony Mannuguerra, Charles Mazeaud, Michael Peres; **Nantes:** Gilles Blancho, Julien Branchereau, Diego Cantarovich, Agnès Chapelet, Jacques Dantal, Clément Deltombe, Lucile Figueres, Raphael Gaisne, Claire Garandeau, MG, Caroline Gourraud-Vercel, Clarisse Kerleau, Christophe Masset, Aurélie Meurette, Simon Ville, Christine Kandell, Anne Moreau, Karine Renaudin, Florent Delbos, Alexandre Walencik, Anne Devis. **Nice:** Laetitia Albano, Damien Ambrosetti, Nadia Ben Hassen, Mathilde Blois, Marion Cremoni, Matthieu Durand, Patricia Goldis, Clément Gosset, Fatimaezzahra Karimi, Antoine Sicard, Giorgo Toni. **Barcelona:** Irina Torres, Maria Meneghini, Delphine Kervella, Joana Sellares, Manel Perelló, José Zúñiga.

## Supplementary Material

SUPPLEMENTARY FIGURE S1
**(A)** Supplementary Figure S1 Distribution of time on dialysis after Propensity score matching in the living donor and brain death donor cohorts. **(B)** Distribution of time on dialysis after Propensity score matching in the living donor and controlled donor cohorts after circulatory death cohorts.
