# Supplementary figures and images for: Benefits of Living Over Deceased Donor Kidney Transplantation in Elderly Recipients. A Propensity Score Matched Analysis of a Large European Registry Cohort
Source: Transpl Int. 2024 Aug 23;37:13452. doi: 10.3389/ti.2024.13452 (PMC11387891; doi:10.3389/ti.2024.13452)

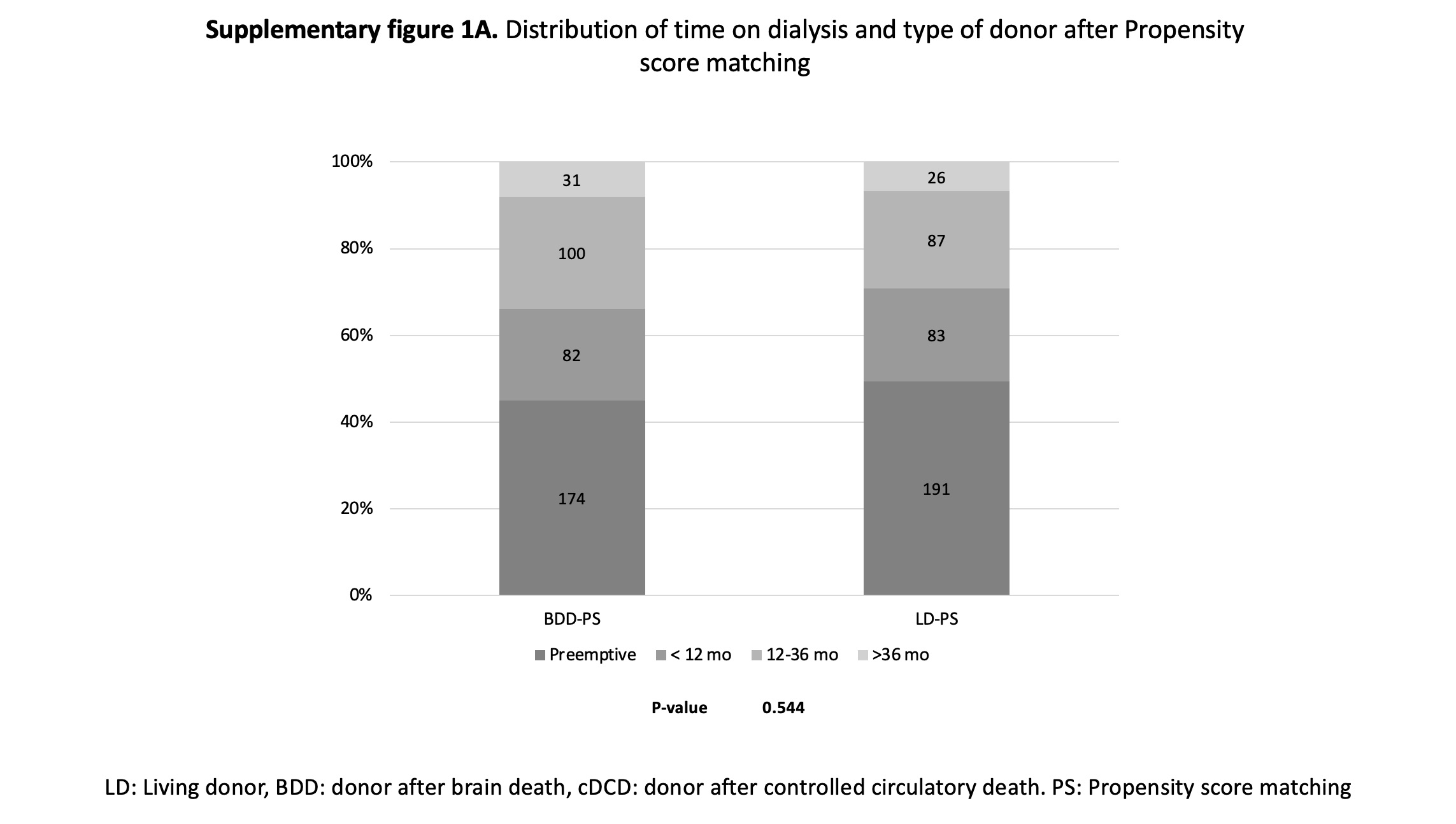

Supplement: Supplementary file 1 [file Image1.JPEG]

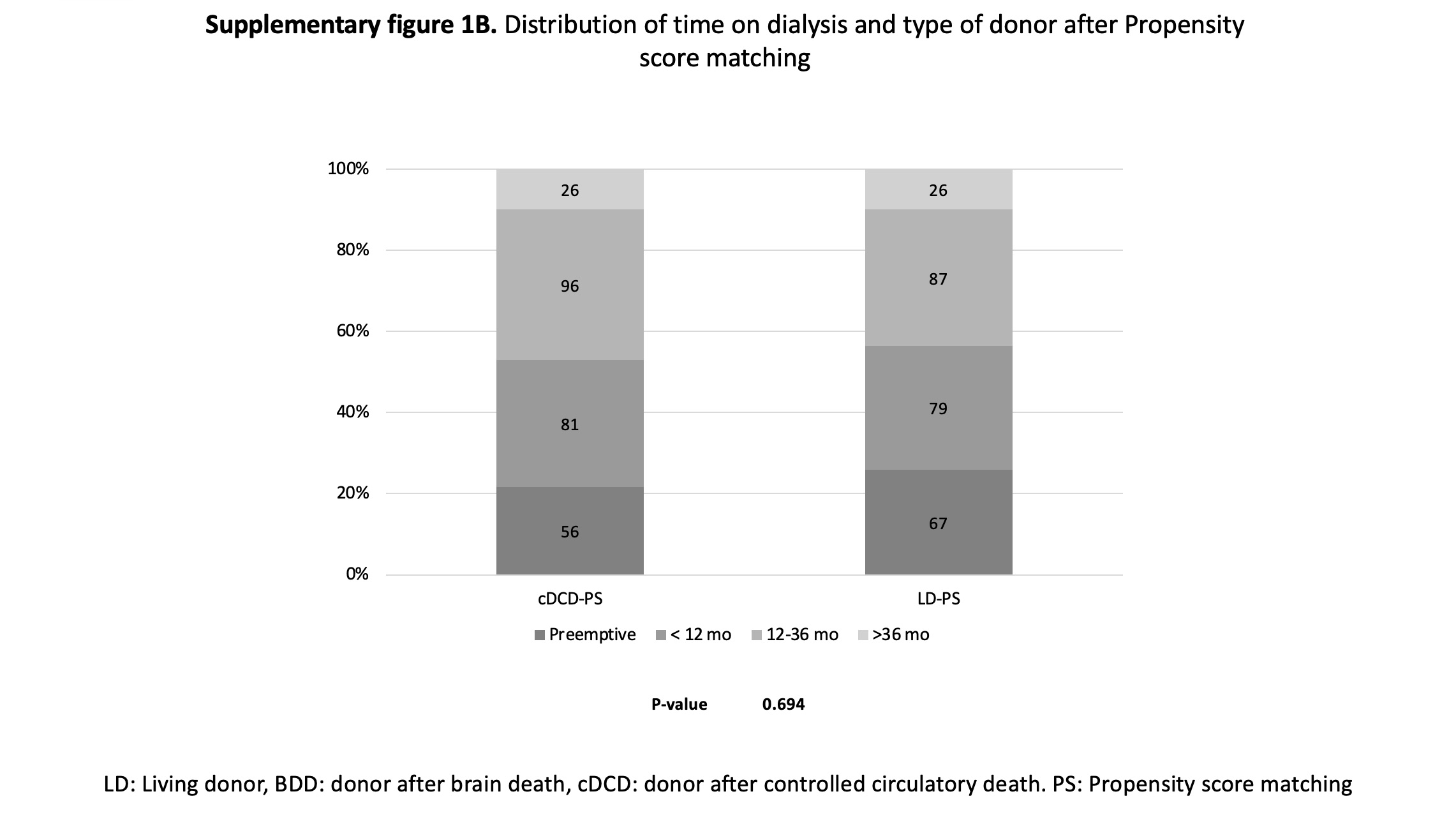

Supplement: Supplementary file 2 [file Image2.JPEG]
